# Stratification of diabetes in the context of comorbidities, using representation learning and topological data analysis

**DOI:** 10.1038/s41598-023-38251-1

**Published:** 2023-07-16

**Authors:** Malgorzata Wamil, Abdelaali Hassaine, Shishir Rao, Yikuan Li, Mohammad Mamouei, Dexter Canoy, Milad Nazarzadeh, Zeinab Bidel, Emma Copland, Kazem Rahimi, Gholamreza Salimi-Khorshidi

**Affiliations:** 1 Deep Medicine, Oxford Martin School, University of Oxford, Oxford, UK; 2 Mayo Clinic Healthcare, 15 Portland Place, London, UK; 3 Nuffield Department of Women’s and Reproductive Health, Medical Science Division, University of Oxford, Oxford, UK

**Keywords:** Diseases, Health care

## Abstract

Diabetes is a heterogenous, multimorbid disorder with a large variation in manifestations, trajectories, and outcomes. The aim of this study is to validate a novel machine learning method for the phenotyping of diabetes in the context of comorbidities. Data from 9967 multimorbid patients with a new diagnosis of diabetes were extracted from Clinical Practice Research Datalink. First, using BEHRT (a transformer-based deep learning architecture), the embeddings corresponding to diabetes were learned. Next, topological data analysis (TDA) was carried out to test how different areas in high-dimensional manifold correspond to different risk profiles. The following endpoints were considered when profiling risk trajectories: major adverse cardiovascular events (MACE), coronary artery disease (CAD), stroke (CVA), heart failure (HF), renal failure (RF), diabetic neuropathy, peripheral arterial disease, reduced visual acuity and all-cause mortality. Kaplan Meier curves were plotted for each derived phenotype. Finally, we tested the performance of an established risk prediction model (QRISK) by adding TDA-derived features. We identified four subgroups of patients with diabetes and divergent comorbidity patterns differing in their risk of future cardiovascular, renal, and other microvascular outcomes. Phenotype 1 (young with chronic inflammatory conditions) and phenotype 2 (young with CAD) included relatively younger patients with diabetes compared to phenotypes 3 (older with hypertension and renal disease) and 4 (older with previous CVA), and those subgroups had a higher frequency of pre-existing cardio-renal diseases. Within ten years of follow-up, 2592 patients (26%) experienced MACE, 2515 patients (25%) died, and 2020 patients (20%) suffered RF. QRISK3 model’s AUC was augmented from 67.26% (CI 67.25–67.28%) to 67.67% (CI 67.66–67.69%) by adding specific TDA-derived phenotype and the distances to both extremities of the TDA graph improving its performance in the prediction of CV outcomes. We confirmed the importance of accounting for multimorbidity when risk stratifying heterogenous cohort of patients with new diagnosis of diabetes. Our unsupervised machine learning method improved the prediction of clinical outcomes.

## Introduction

Diabetes mellitus (DM) affects 422 million people worldwide, and 1.5 million deaths are directly attributed to diabetes and its complications each year^1^. DM is increasingly recognised as a highly heterogeneous disease with varying clinical manifestations, trajectories, and ranges of complications^2^. Identifying various phenotypes early in the diagnostic process, while consequences may still be avoidable, may allow for a more individualised approach to therapy and potentially improve clinical outcomes.

A wide spectrum of approaches and strategies has been employed to characterise different pathophysiological and clinical phenotypes of diabetes, including emerging approaches integrating genomics, metabolomics, biomarkers, physiology, and behavioural medicine, to name a few^3^. On the other hand, the growing popularity and adoption of electronic health records (EHR), together with advances in machine learning (ML), including deep learning (DL), have opened up unprecedented opportunities for characterising disease phenotypes, particularly in the presence of multimorbidity^4^. DL architectures have shown superior results, compared to their simpler counterparts^5^, when dealing with EHR (i.e., large-scale and complex longitudinal mixed-type data containing “concepts” such as diagnoses, medications, measurements, and more). This is partly due to such models’ ability to learn valuable representations from raw (or minimally processed) data, instead of being given such representations by experts. For instance, BEHRT, a Transformer-based DL architecture^6^, which has shown superior performance in a range of EHR-based prediction tasks, turns EHR concepts into “representations” or “embeddings” (a numeric vector) that attempt to capture both their broader clinical meanings and their specific context in a given point in a patient’s record; the latter property is what makes such representations “contextual”. In other words, two different instances of diabetes (in the same patient or two different patients) can be mapped to two different representations^7^.

In this study, we employed a topological data analysis (TDA) technique called Mapper^8^ to assess that the contextual representation of diabetes derived from BEHRT contains the necessary information for phenotyping patients. Mapper allows studying how similarities of each patient’s contextual embedding of incident diabetes correspond to similarities in their future health trajectories. In this context, the TDA can help us understand the shape of such a “disease manifold” and find regions that show similar forward trajectories^9,10^.

**Figure 1:**
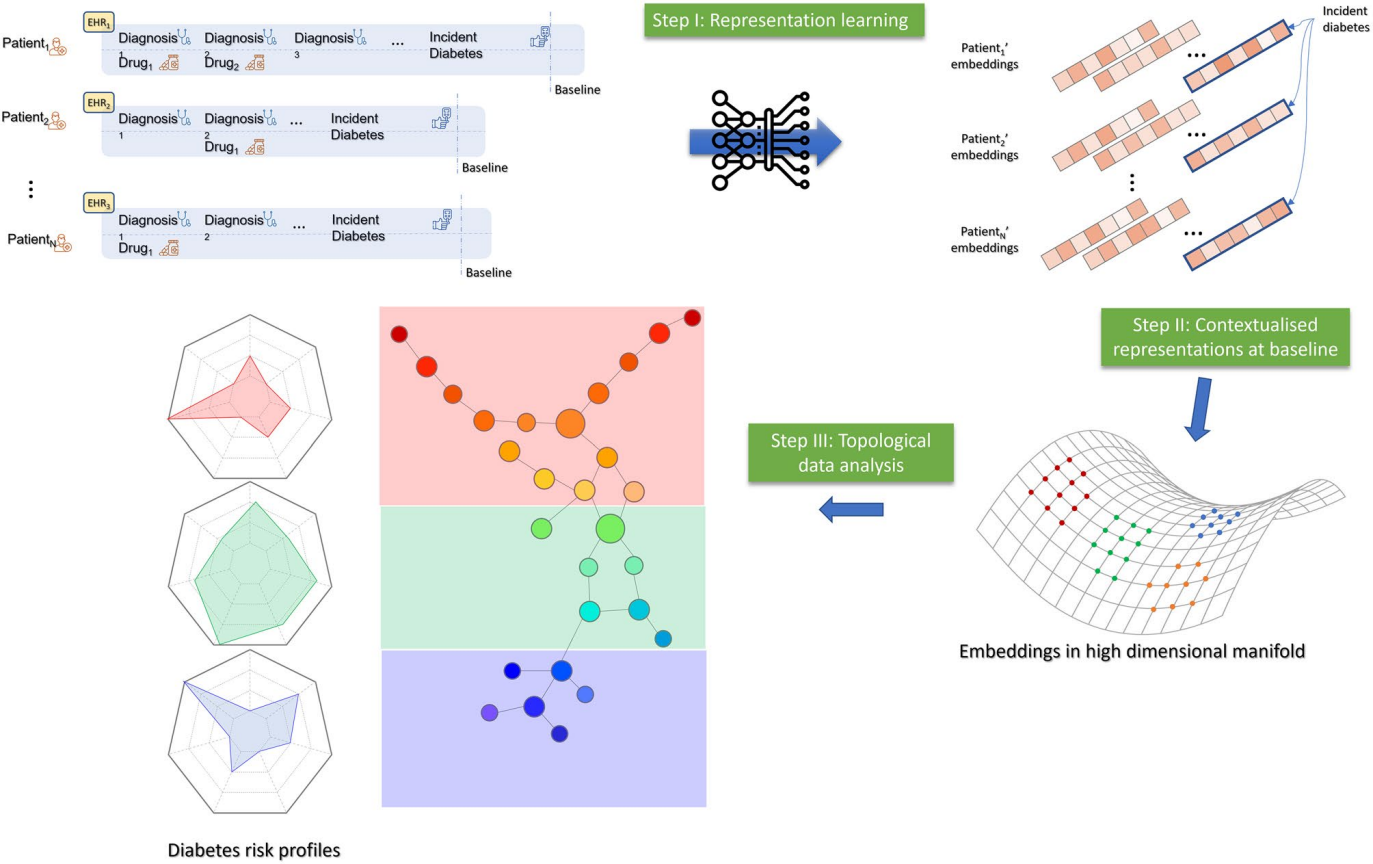
An overview of our investigative approach. (**A**) The process starts with EHR, where the baseline (T_0_) is defined as the date of incident diabetes for each patient. The data before the baseline is fed to BEHRT for learning the contextual representation of each patient’s incident diabetes; the data after the baseline are used for profiling the patients’ risk trajectories. (**B**) Each patient is summarised by their incident diabetes embeddings (i.e., 120-dimensional vectors). (**C**) A point cloud, where each point is a patient, is formed for TDA, which aims to mine the diabetes manifold. (**D**) Using TDA, these points are analysed, and homogeneous regions are mined and profiled. Note that each circle in the TDA graph consists of multiple patients.

In other words, we explore using contextual embeddings resulting from BEHRT to stratify a highly heterogenous group of multimorbid patients at the onset of their diabetes diagnosis. We then process these embeddings using TDA to derive the “diabetes manifold”^11–13^, a map where different regions correspond to different clinically meaningful diabetes phenotypes. We validated our results by profiling the patients using risk factors, associated comorbidities, and clinical outcomes, including their progress towards developing cardiovascular, renal, and other microvascular complications (Fig. 1).

## Materials and methods

### Data and study population

This study has been carried out using linked electronic health records from the CPRD (Clinical Practice Research Datalink^14,15^) collected between 1985 to 2015 from a network of GP practices across the UK. All methods were performed by the relevant guidelines and regulations, and all experimental protocols were approved by CPRD’s Research Data Governance (RDG) Process. CPRD never receives information that identifies patients and only provides anonymised health data to approved researchers. Patients registered with GP practices may opt out of his/her information being shared for research, but individual consent is not required. CPRD is linked to other national administrative databases including hospitalisations (Hospital Episode Statistics), death registration (Office of National Statistics), and the Index of Multiple Deprivation, which makes the CPRD database a comprehensive resource for prospective analysis of UK primary care data. It encompassed 60 million patients, including 16 million registered patients, providing one of the largest EHR databases in the world. It contains data regarding demographics, diagnoses, therapies, and tests. CPRD has ethical approval from the Health Research Authority to support research using anonymised patient data. Many studies have demonstrated the utility of CPRD in establishing detailed clinical phenotypes^5,16–18^. The hospital encounters are provided with the corresponding ICD-10 code (International Classification of Diseases-10th Edition), whereas the general practitioner encounters are supplied with the corresponding Read code^19,20^. For this study, both these codes are mapped to CALIBER codes^21^, which provides a clinically meaningful classification of diseases.

In this study, we used the same coding system of the original BEHRT paper; as such, diabetes mellitus (DM) is defined as per its CALIBER code, which combines the three main subtypes: diabetes type 1 (T1DM), type 2 (T2DM) and unclassified diabetes^21^. However, in the second part of the analysis, we show to what extent our algorithm could distinguish between these subtypes. Similarly, comorbid conditions were also defined based on their CALIBER codes, including Read codes (for primary care diagnoses), ICD10 codes (for secondary care diagnoses) and OPCS4 (for secondary care procedures). Causes of death from the death registry were also used when relevant, as per CALIBER.

### Representation learning and topological data analysis (TDA)

BEHRT has shown superior performance in a range of risk prediction tasks compared to other ML/DL models. Unlike most of its counterparts, the representations learned by BEHRT are contextual. Thus, instead of learning a single representation of diabetes, BEHRT can learn a unique representation for each instance of diabetes. Depending on the context of a given example of diabetes (e.g., patient’s other morbidities, medications), its corresponding embedding can differ (Sect. 2 in the Supplementary Materials). A TDA technique called Mapper^8^ was then used to analyse differences in various areas in the high-dimensional manifold underlying these embeddings and test whether those areas correspond to distinct risk profiles (Sect. 3 in Supplementary Materials). BEHRT’s optimal hyperparameters were tuned using Bayesian optimisation. This includes the length of embedding, which was found to be optimal at 120. In other words, the “point cloud” for TDA will consist of 9967 diabetes embeddings, each of length 120; each expected to summarise its corresponding patient’s diabetes at the baseline sufficiently. TDA results in a graph where each node represents a group of patients. We partition this graph using multilevel k-way partitioning^23^ as implemented by the METIS graph partitioning software^24^ to derive meaningful diabetes phenotypes. A high-level view of the process (from EHR to phenotypes) is outlined in Fig. 1. The resulting phenotypes can be characterised by age, gender, BMI distributions, and prevalence of hypertension, arterial fibrillation, chronic kidney disease, and high cholesterol at baseline (Sect. 4 in Supplementary Materials).

### Study design and inclusion criteria

We have extracted data related to multimorbid DM patients. To have broad coverage of patients’ medical history and similarly to BEHRT^22^, only individuals with at least five visits in their records are considered in this study. Moreover, to provide our models with enough past events to learn from, we included only those patients whose diagnosis of DM was registered at least as *nth* comorbidity (i.e., DM occurrence is only considered if at least *n*−1 other disease was registered before it). For example, in this analysis, we used n = 7, which is large enough to enable the model to learn from many past events and produce a significant number of patients. This criterion identified 9967 DM patients for inclusion in our analysis. Reducing *n* significantly would make it hard for the model to pick up a meaningful "context” of patients. Meanwhile, increasing it significantly would increase the age and the comorbidities of our study population significantly and increase the risk of introducing bias in the results. For these patients, we have computed the contextual embeddings corresponding to their incident DM at baseline, defined as the first occurrence of diabetes that happened as the 7th comorbidity.

### Clinical outcomes

The following endpoints were included: composite major adverse cardiovascular events: cardiovascular death, coronary artery disease, stroke, heart failure (MACE), coronary artery disease (CAD), stroke, heart failure (HF), renal failure (RF), diabetic neuropathy, peripheral arterial disease, reduced visual acuity and all-cause mortality. These endpoints were defined using disease and procedures provided by CALIBER^21^. To analyse all clinical outcomes, patients were censored at the end of follow-up (10 years after entering the study), when lost to follow-up or if they died.

### Statistical analysis

Baseline characteristics and the prevalence of comorbidities among 9967 included patients were described when they were included in the study, which was defined as the time of new-onset diabetes. Kaplan Meier (KM) curves were plotted for each derived subtype for all endpoints. P-values for the probability of each clinical outcome in the four identified phenotypes were obtained using a multivariate log rank test^25,26^. All survival analyses were performed using the lifelines Python package^27^. Areas under the receiver operating curves (AUC) were calculated for prediction risk of MACE in the follow-up period using the established QRISK model alone and augmented with TDA predictors. All predictors used in QRISK3 were included in computing the AUCs (see legend in Fig. 5). For comparison, AUCs were also produced for TDA-derived predictors and when augmenting the QRISK3 model with these TDA predictors. Only patients with complete data are considered in the denominator when producing counts for variables with missing data (such as BMI; Sect. 5 in Supplementary material).

### Ethics

As described in the publication by Wolf et al. Titled: Data Resource profile: CPRD: “CPRD obtains annual research ethics approval from the UK’s Health Research Authority (HRA) Research Ethics Committee (REC) (East Midlands—Derby, REC reference number 05/MRE04/87) to receive and supply patient data for public health research. Therefore, no additional ethics approval is required for observational studies using CPRD data for public health research, subject to individual research protocols meeting CPRD data governance requirements.”

## Results

### Stratification of patients with the diagnosis of diabetes using the BEHRT-TDA model

**Figure 2:**
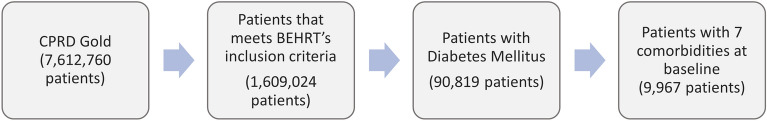
Flow chart showing key steps to reach our final cohort of patients.

**Figure 3:**
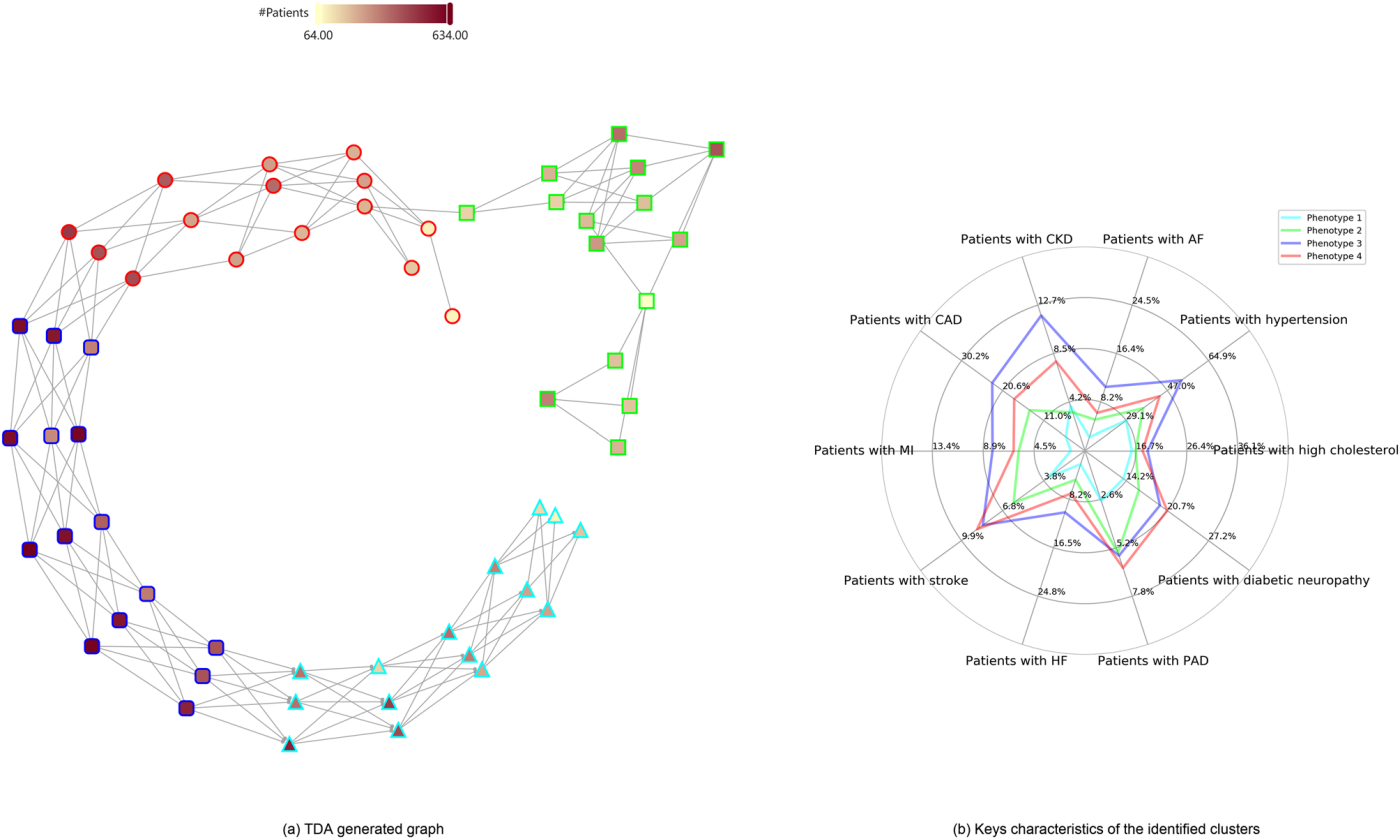
Diabetes phenotypes identified using TDA. (**a**) The network resulting from TDA, where each node contains patients that have similar diabetes embeddings. (**b**) The distribution of known cardiovascular risk factors and pre-existing diseases within the four identified regions/subtypes. In the TDA network, nodes correspond to patients with similar representation; nodes are connected if they share at least one patient (see “Materials and methods”). The nodes and their colours represent the 4 clusters obtained using the METIS partitioning method. A fully interactive version of this figure that shows further details at the node and cluster level is available at: https://deepmedicine.github.io/TDA/multiple.html.

We analysed the EHR of 9967 patients registered between 1985 and 2015. Among the total number of DM cases included in this study, type 1 diabetes and other diabetes type represented 5.9% (585) and 5.2% (514), respectively. 93% (9266) were classified as type 2 diabetes. Figure 2 presents the flow chart of the study. Figure 3a shows the representative graph of the cohort of patients with a new diagnosis of diabetes, in which each node denotes patients with similar clinical characteristics. An interactive version of this graph is provided along with Fig. 3b, which shows the radar plot visualisation of the distribution of comorbidities among these four identified phenotypes.

### Baseline characteristics of four identified distinct phenotypes

Baseline characteristics of patients included in these four phenotypes at the time of a new diagnosis of diabetes have been described in Table 1. 51.4% of all included patients were male with a mean (SD) age of 65.5 (12.9) years. Compared with phenotypes 1 and 2, patients classified into phenotypes 3 and 4 were older (mean age in years (SD): phenotype 1—63 (13), phenotype 2—62 (12), phenotype 3—68 (12) and phenotype 4—67 (12)), more likely to have diabetes type 1 (phenotype 1—4%, phenotype 2—4%, phenotype 3—6% and phenotype 4—9%), to be on insulin (phenotype 1—2%, phenotype 2—2%, phenotype 3—4% and phenotype 4—7%), had a previous history of atrial fibrillation (phenotype 1—2.8%, phenotype 2—5%, phenotype 3—9% and phenotype 4—7.5%) and chronic kidney disease (phenotype 1—4%, phenotype 2—3%, phenotype 3—11% and phenotype 4—9%). There was, however, no significant difference in body mass index (BMI), total cholesterol level, systolic blood pressure and history of smoking among the four subgroups.

**Table 1:** Baseline characteristics of four identified subgroups of patients with diabetes.

	Phenotype 1	Phenotype 2	Phenotype 3	Phenotype 4	All patients
Number of patients	2587	1885	4461	2460	9967
Mean age (SD)	63.6 (13.4)	62.2 (12.9)	68.4 (12.0)	67.1 (12.4)	65.6 (12.9)
% Men (N)	45.8% (1185)	55.8% (1052)	51.9% (2314)	51.4% (1265)	51.40% (5123)
Mean weight (SD)	87.3 (21.2)	90.9 (22.1)	87.6 (20.3)	88.3 (20.7)	88.5 (21.0)
Mean BMI (SD)	31.2 (6.9)	31.7 (6.9)	31.2 (6.5)	31.4 (6.8)	31.4 (6.7)
Mean systolic blood pressure (SD)	140.54 (17.53)	139.77 (17.75)	139.85 (18.18)	138.70 (18.59)	139.66 (17.98)
% Patients using insulin (N)	2.16%	2.33%	4.37%	6.87%	3.96%
% Patients with T1DM (N)	4.0% (103)	4.1% (77)	6.1% (270)	8.8% (217)	5.9% (585)
% Patients with T2DM (N)	93.2% (2412)	92.5% (1743)	94.2% (4202)	91.2% (2244)	93.0% (9266)
% Patients with other DM (N)	5.5% (141)	5.7% (107)	4.2% (189)	5.9% (144)	5.2% (514)
% History of high cholesterol (N)	15.9% (412)	14.7% (277)	18.7% (832)	17.4% (427)	15.9% (412)
% History of atrial fibrillation (N)	2.8% (72)	5.2% (98)	9.0% (403)	7.5% (184)	2.8% (72)
% History of CKD (N)	4.1% (105)	3.1% (59)	11.0% (490)	8.9% (218)	4.1% (105)
% History of smoking (N)	49.6% (1269)	51.6% (957)	51.6% (2275)	54.1% (1310)	52.03% (5114)
Top diseases at baseline Percent of patients (N)					
Dorsalgia	55.1% (1425)	44.6% (840)	46.4% (2072)	47.5% (1169)	48.5% (4832)
Other soft tissue disorders, not elsewhere classified	52.3% (1353)	42.8% (806)	46.9% (2092)	45.7% (1125)	47.2% (4703)
Essential (primary) hypertension	35.0% (905)	33.6% (634)	51.1% (2279)	43.0% (1057)	41.3% (4114)
Other joint disorders, not elsewhere classified	43.6% (1127)	35.6% (671)	37.7% (1684)	36.7% (904)	38.6% (3851)
Unspecified acute lower respiratory infection	38.1% (986)	29.2% (551)	34.0% (1518)	34.4% (846)	34.3% (3422)
Acute upper respiratory infections of multiple and unspecified sites	35.1% (909)	24.3% (458)	25.8% (1152)	25.0% (615)	27.5% (2742)
Mental and behavioural disorders due to use of tobacco	22.9% (593)	22.7% (427)	18.6% (831)	20.1% (494)	21.0% (2089)
Obesity	19.8% (512)	21.3% (402)	19.6% (873)	20.4% (503)	20.1% (1999)
Chronic rhinitis, nasopharyngitis and pharyngitis	25.0% (647)	18.8% (355)	15.6% (698)	16.4% (404)	18.8% (1877)
Other arthrosis	18.6% (480)	17.2% (324)	19.1% (853)	18.1% (446)	18.5% (1839)
Top drugs at baseline Percent of patients (N)					
Antibacterial drugs	95.7% (2475)	90.9% (1714)	93.0% (4150)	93.1% (2290)	93.3% (9303)
Analgesics	84.8% (2194)	79.1% (1491)	85.2% (3802)	86.3% (2122)	84.2% (8393)
Drugs used in rheumatic diseases and gout	86.9% (2247)	78.0% (1471)	81.2% (3621)	82.0% (2016)	82.2% (8194)
Topical corticosteroids	65.7% (1699)	55.2% (1041)	61.2% (2732)	61.8% (1520)	61.3% (6106)
Hypertension and heart failure	32.4% (839)	43.4% (819)	74.7% (3331)	68.7% (1689)	57.3% (5707)
Diuretics	39.6% (1025)	39.2% (739)	67.6% (3014)	63.1% (1552)	53.3% (5315)
Antisecretory drugs and mucosal protectants	49.2% (1274)	49.3% (929)	53.4% (2384)	57.0% (1401)	52.7% (5254)
Vaccines and antisera	52.1% (1348)	43.3% (817)	54.3% (2421)	51.7% (1271)	51.0% (5087)
Lipid-regulating drugs	32.7% (846)	38.4% (724)	60.7% (2707)	62.9% (1548)	50.8% (5065)
Nitrates, calcium-channel blockers & other antianginal drugs	35.1% (909)	35.2% (663)	65.4% (2918)	58.5% (1440)	49.7% (4950)
Previous diagnosis of outcomes of interest					
Coronary artery disease	7.7% (200)	14.2% (268)	23.4% (1042)	21.7% (534)	17.5% (1741)
Stroke	4.3% (111)	5.7% (107)	8.5% (381)	9.3% (230)	7.1% (705)
Heart failure	0.6% (16)	3.2% (60)	5.8% (258)	5.5% (135)	4.1% (404)
Renal failure	4.4% (115)	4.4% (82)	12.1% (539)	10.0% (245)	8.3% (823)
MACE	12.1% (314)	21.0% (396)	33.1% (1477)	31.9% (785)	25.4% (2534)

*BMI* body mass index, *T1DM* type 1 diabetes mellitus, *T2DM* type 2 diabetes mellitus, *CKD* chronic kidney disease, *MACE* major cardiovascular events.

*Phenotype 1 (young with chronic inflammatory conditions)* could be characterised by the younger age of included patients who frequently presented with dorsalgia and chronic inflammatory conditions and had the overall lowest risk of pre-existing cardiovascular and renal diseases. *Phenotype 2 (young with CAD)* included relatively younger DM patients who more frequently had CV risk factors or pre-existing diagnosis of CAD. Patients in phenotypes 3 and 4 were relatively older and had a higher frequency of pre-existing cardio-renal diseases (HF 5%, MACE 32%, CAD 22%). *Phenotype 3 (older with hypertension and renal disease)* could be further distinguished by the highest percentage of patients with pre-existing diagnoses of HTN and RF at the time of inclusion into the study. In contrast, patients in *phenotype 4 (older with previous CVA)* had the highest percentage of patients with a prior history of CVA. They were more frequently started on insulin at the time of diagnosis of diabetes.

### Top ten comorbidities and most frequently prescribed drugs at baseline

At baseline, 4832 out of 9967 (48%) patients newly diagnosed with diabetes had a history of severe back pain, 4703 (47%) soft tissue disorders and 3851 (38%) joint disorders, and 1999 (20%) were diagnosed with obesity. Hypertension (HTN) was one of the most common comorbidities among DM patients (4114 (41%) with the highest percentage of 2279 (51.1%) in phenotypes 3 and 4). At the time of the new diagnosis of diabetes, antibiotics (93%), analgesia (84%), drugs used for rheumatic disorders and gout (82%) and topical corticosteroids (61%) followed by antihypertensive and HF medicines (57%) were most frequently present on prescriptions of those included in the analysis. Noteworthy, the frequency of pre-existing cardiovascular and renal comorbidities at baseline increased significantly in phenotypes 2, 3 and 4 (Table 1).

### Follow-up and prognosis of four identified phenotypes.

Patients had a follow-up duration of up to ten years with a median follow-up of 6.08 years and a total of 64,000 person-year. Within ten years of follow-up, 2592 patients (26%) experienced a MACE, 2515 patients (25%) died, and 2020 patients (20%) suffered RF. The following percentages of patients within each phenotype have been hospitalised over the ten years of follow-up (phenotype 1—71%, phenotype 2—73%, phenotype 3—76.5%, phenotype 4—78.5%). The new DM diagnosis prompted the initiation of lipid-lowering drugs, drugs used in DM and HTN in all four phenotypes, reaching the highest use in phenotypes 2 and 3. Those two subgroups also had the highest percentages of patients with prescribed drugs used to manage CAD and HF (beta-blockers: 33.42% phenotype 2 and 46.02% phenotype 3; nitrites and calcium channel blockers: 45.52% phenotype 2 and 61.17% phenotype three and antiplatelets: 47.6% phenotype 2 and 59.87% phenotype 3).

### The probability of cardio-renal outcomes in four identified phenotypes

**Figure 4:**
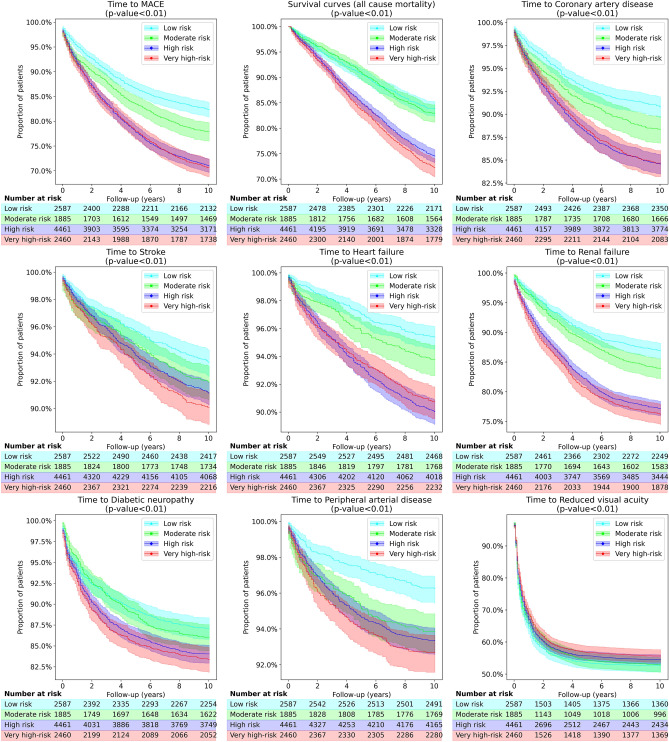
Kaplan–Meier survival curves demonstrate the probability of each clinical outcome in the four identified phenotypes. All clinical outcomes were defined per their CALIBER codes (21). P-values for this analysis are obtained using a multivariate log rank test^25,26^.

Kaplan–Meier survival curves demonstrating the probability of survival for outcomes of interest in each identified phenotype are presented in Fig. 4.

Although the difference in survival curves for all chosen clinical outcomes was significant among four phenotypes identified by our model, the differentiation was least evident for diabetic neuropathy, reduced visual acuity and stroke. Median survival time to MACE and CAD diagnosis over ten years follow-up did not differentiate between phenotypes 3 and 4, which had significantly higher risks of all-cause mortality and cardio-renal outcomes than phenotypes 1 and 2. For RF and all-cause mortality outcomes, there was a split between four phenotypes with significantly lower median survival probability in phenotypes 1 and 2 compared to phenotypes 3 and 4. Phenotype 4 had the highest probability of all-cause mortality, CVA, RF, PAD and diabetic neuropathy outcomes.

### The additive role of TDA features in identifying patients with increased risk of MACE

**Figure 5:**
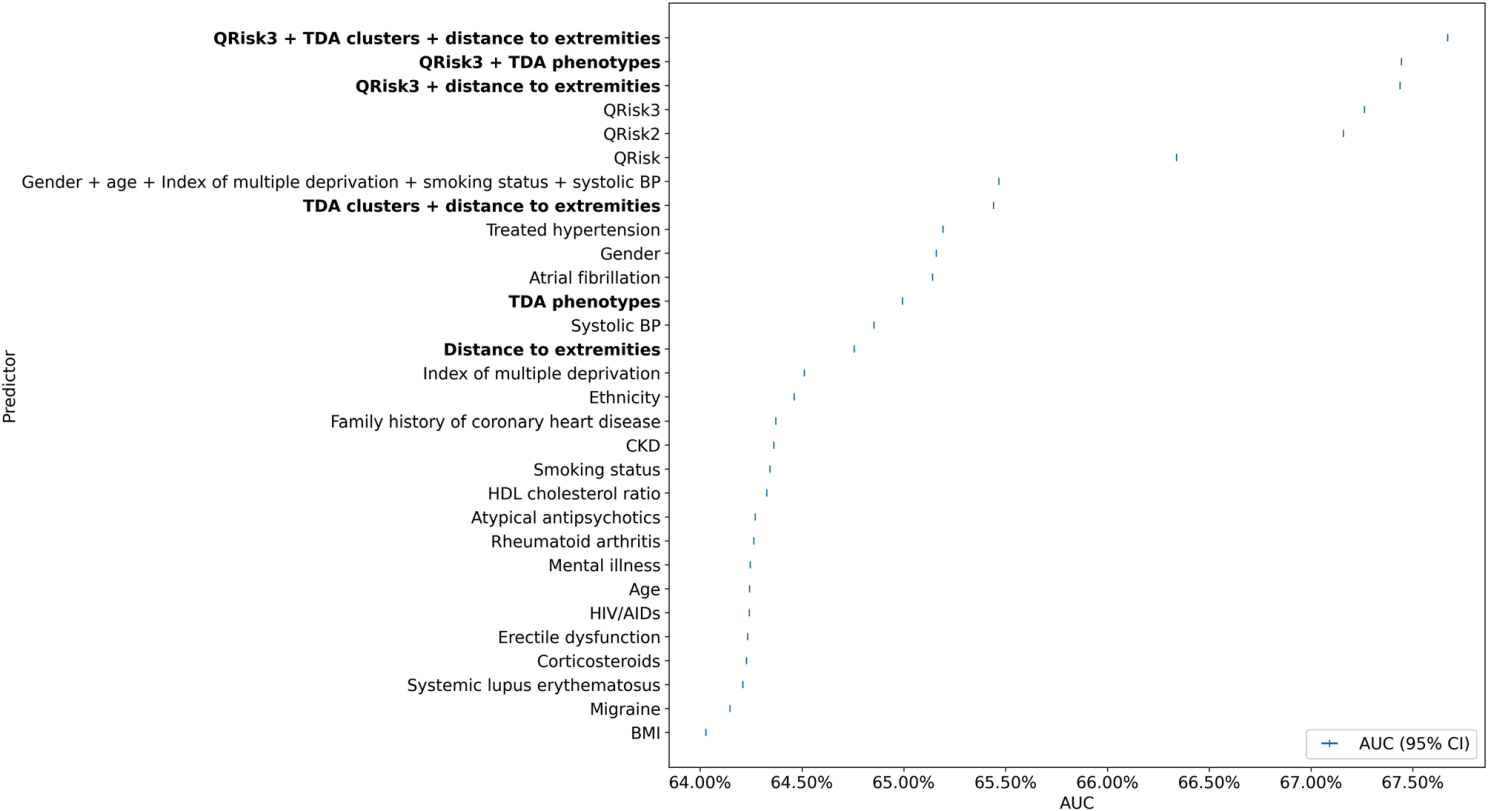
AUC assessed the risk of developing MACE within ten years of DM diagnosis. QRISK, QRISK2 and QRISK3 of developing a MACE within the ten years following the DM diagnosis. All the individual predictors used in the QRISK3 model were included in our analysis in addition to the predictors derived from our TDA analysis (such as distance to extremities, TDA phenotypes, TDA clusters and the distance to extremities). The list of other predictors includes the following: body mass index (BMI), migraine, systemic lupus erythematosus, corticosteroids, erectile dysfunction, HIV/AIDS, mental illness, rheumatoid arthritis, atypical antipsychotics, HDL cholesterol ratio, smoking status, chronic kidney disease, family history of coronary artery disease, ethnicity, index of deprivation, systemic blood pressure, atrial fibrillation, gender, treated hypertension. The AUC was calculated for these models and models using individual predictors of QRISK3. The results show that augmenting QRISK3 with TDA features significantly increased the AUC (to an extent larger than the one obtained when upgrading from QRISK2 to QRISK3). Moreover, TDA features score better than all individual predictors of QRISK3. Even when taken individually, they score better than most QRISK3 individual predictors. The predictive value of the QRISK3 model also improves by adding two features derived from the TDA graph: 1) the TDA-derived phenotype and 2) the distances to both extremities of the TDA graph (as defined by Dijkstra's algorithm).

Figure 5 compares areas under the receiver operating curves (AUC) for various predictors and several QRISK models assessing the CVD risk. QRISK, QRISK 2 and QRISK 3 and models integrating TDA features with QRISK assessments in predicting the risk of MACE within ten years from the diagnosis of diabetes (and inclusion in the study). QRISK3 model’s AUC was augmented from 67.26% (CI 67.25–67.28%) to 67.67% (CI 67.66–67.69%) by adding two features derived from the TDA graph: specific TDA-derived phenotype and the distances to both extremities of the TDA graph (this improvement is by an extent which is larger than the one obtained when upgrading from QRISK2 (0.6716) to QRISK3 (0.6725)). Moreover, the TDA features had higher predictive values than all individual predictors of QRISK3.

## Discussion

In this study, we demonstrated the utility of an unsupervised machine learning BEHRT-TDA method in stratifying a highly heterogenous group of DM patients using an example of a cohort diagnosed with diabetes as the seventh’s comorbidity. First, by leveraging contextual embedding with topological data analysis in a novel way, we developed and validated a model allowing us to study diabetes in the multimorbidity context. Second, we identified four distinct phenotypes of patients and confirmed their differential baseline characteristics at the time of new-onset diabetes. Third, we confirmed their distinct disease progression over time by analysing their cardiovascular, renal, and other microvascular outcomes. Lastly, we showed that features derived from TDA, when added to a known CV risk model called QRISK, can improve the prediction of CV outcomes in DM patients with comorbidities. Our analysis proves that deep learning can be used for multiscale phenotyping of diabetes in the context of multimorbidity.

Diabetes is regarded as a highly heterogeneous disease with significant variation among clinical subtypes and a wide range of patterns of disease progression^28^. As a chronic condition, diabetes frequently occurs in the context of other diseases, which cumulatively have a more substantial impact on a patient’s quality of life and the risks of developing severe complications and mortality than when diabetes is considered in the separation^29–31^. The consensus report from the American Diabetes Association (ADA) and the European Association for the Study of Diabetes (EASD) in 2020 emphasised the personal approach to managing DM patients. It encouraged disease phenotyping and risk stratification, especially when considering novel glucose-lowering therapies^32^. Current clinical guidelines suggest stratification of DM patients into low, moderate, high, and very high-risk groups based on their CV risk profile and provide evidence for using CV risk factors targeted therapies in those classified as high-risk groups^33^. Such an approach stresses the role of cardiometabolic precursors in the overall mortality. Still, it does not fully consider the impact of higher multimorbidity burden, which includes the number of concordant and discordant conditions, the longer duration of living with multimorbidity, polypharmacy and the higher risk of associated adverse events. Our methodology derived from unsupervised deep learning considers not only the multitude of variabilities but, most importantly, looks at diabetes in the context of other comorbidities and offers an alternative approach to previously described clustering methods. We classified patients into four subgroups based on the regions of the TDA networks. Similarly to the stratification of DM patients based on pre-existing CVD, we have shown the correlation between an increased prevalence of pre-existing CV and renal disease in phenotypes 3 and 4. We identified that those two subgroups included more patients with T1DM and T2DM treated with insulin. It is in line with general observations that with early onset DM, longer duration, and the advanced stage of the disease, insulin-dependent T2DM is associated with a worse prognosis, higher risk of complications and an increased death rate.

A recent review paper summarises studies using various methodologies to cluster T2DM in the setting of multimorbidity. It concludes that age and deprivation are the leading drivers of multimorbidity in DM patients. In our study, using ML-derived phenotyping, older age was a proxy for multimorbidity and a higher prevalence of cardio-renal disease at baseline. It defined the future outcomes in our cohort of DM patients. The probability of cardio-renal outcome differed between younger patients classified to phenotypes 1 (young with chronic inflammatory conditions) and 2 (young with CAD) when compared to relatively older patients in phenotype 3 (older with hypertension and renal disease) and phenotype 4 (older with previous CVA). The prevalence of cardio-renal disease was also higher in phenotypes 3 and 4 (among relatively older patients), which may suggest that biological rather than chronological age would be more accurate in determining the risk of mortality.

Diabetes rarely presents without comorbidities. We identified musculoskeletal diseases and HTN as the most frequent conditions in all four subgroups. Such conditions as dorsalgia and other musculoskeletal diseases are frequently related to physical inactivity leading to obesity, recognised as an essential risk factor for the development of T2DM. In this cohort, we observed a significantly higher prevalence of musculoskeletal conditions (50%) than obesity (20%). Additionally, on average, 41% of DM patients had HTN, and 57% were treated with medications recognised as treatments for HTN and HF. A large meta-analysis of randomised controlled trials utilising various BP-lowering strategies has recently provided evidence of the causal relationship between elevated BP and the risk of T2DM^34^—https://pubmed.ncbi.nlm.nih.gov/34774144/. The results demonstrated that BP-lowering by five mmHg reduced the risk of diabetes by 11%, comparable to the well-established cardioprotective effect of antihypertensive therapy. In the current study, we showed a higher prevalence of HTN in subgroups of DM people with the highest future risk of renal and cardiovascular complications and increased mortality. It highlights the importance of early screening for signs of heart and kidney disease in hypertensive patients diagnosed with diabetes. It supports the initiation of novel glucose-lowering therapies with cardio-renal benefits as their first treatment line.

One of the main objectives of disease phenotyping is to derive meaningful subgroups in the data that show similarities in both representation and clinical outcomes. Such segmentations have been previously achieved through clustering techniques applied to expert-defined representations. In this study, however, we explored combining learned contextual representations with TDA to introduce a novel alternative for mining latent structures in complex high-dimensional data. TDA attempts to identify the underlying “shape” of the data^35^ and is often perceived as an alternative to both algebraic methods, which are often too rigid to deal with complex data, and to clustering methods that usually require setting thresholds and producing output which is too discrete. TDA has been previously applied to answer biomedical research questions^11–13,36,37^. Our study confirms those previous reports that TDA has the potential to characterise DM phenotypes in the context of multimorbidity. Our results suggest that the manifold underlying these representations/embeddings shows distinct neighbourhoods, which can provide policymakers and medical guidelines with tools for stratifying and segmenting large, diverse patient populations. Lastly, this study explored the utility of TDA and contextual embedding to identify subgroups. We demonstrated that adding TDA features to the QRISK score significantly improves model performance. Unsurprisingly, our unsupervised ML method outperformed a CV risk prediction statistical model.

Further interventional studies are needed to confirm whether such classification can guide pharmacotherapy with novel glucose-lowering treatments offering cardiovascular and renal protection as part of the personalised therapy. Although other groups have previously proposed that approach, it has yet to enter clinical practice. If successful, such an approach of using ML and TDA to select subgroups of patients with the highest risk of certain outcomes could justify the development of clinical pathways for early screening, long-term monitoring, and the use of lifelong targeted therapies in those high-risk groups of patients.

Our study has some limitations. First, our method has a few free parameters that need to be empirically set, which may impact its direct applicability. Moreover, the current evaluation is labour-intensive. Early reports may suggest that using so-called knowledge graphs could offer an alternative evaluation tool and improve feasibility. Furthermore, during the cohort selection, we kept only those DM patients who had registered several encounters with their GPs before the DM diagnosis. Although this was an important step to assure the robustness of our method, it might have compromised the model’s generalizability towards the low-risk group (exclusion of subjects with diabetes and no other known comorbidities and those with fewer interactions with health services). Another limitation is the fact that in the survival analysis, we omitted cases with missing data, following the principle of ‘complete case analysis’, which may introduce systematic bias if data are not missing at random and if patients with missing data are systematically different than those included in the analysis. In our cohort, patients with missing data could potentially represent a sicker subgroup of multimorbid DM patients missing follow-up appointments or not engaging with their healthcare providers. Moreover, the current work only uses the limited information available within EHR. The future work will couple EHR with detailed information about lifestyle and environment, available through digital devices and technologies and possibly with genetic analysis. Such blood biomarkers as HbA1c are included in the EHR but were not available in our data set and therefore were omitted from our study. Our approach focused on the impact of comorbidities on the longer-term disease prognosis and did not consider the level of glucose control.

## Conclusions

This study confirmed that our novel ML approach combining contextual embeddings with TDA could be utilised for the deep phenotyping of diabetes in the context of other associated chronic conditions. Our analysis highlights the importance of considering multimorbidity when risk-stratifying patients with a new diagnosis of diabetes. Expanding on previously proposed classification based on CV risk factors alone, such strategy identified cohorts of patients with different disease progression and long-term clinical outcomes. We confirmed that a higher prevalence of HTN in people diagnosed with diabetes is associated with a higher risk of renal and cardiovascular complications and increased mortality. The excess risk of all-cause mortality, renal and cardiovascular outcomes was primarily related to pre-existing conditions. It could be predicted in that population without considering the profile of changes in the HbA1c.

## Supplementary Information

Supplementary Information 1.

Supplementary Information 2.

## Data Availability

The CPRD database used for this study has been approved by an Independent Scientific Advisory Committee (ISAC). The ISAC protocol number for this study is: 16_049. To obtain access to CPRD data, researchers are advised to follow the required procedure on the CPRD website Data Access page (https://www.cprd.com/Data-access). The data supporting this study's findings are available from Clinical Practice Research Datalink (CPRD). The link: https://www.cprd.com/Data explains more in-depth about the nature and accessibility of the data. Furthermore, regarding accessibility, https://www.cprd.com/primary-care explains: “Access to data from CPRD is subject to a full licence agreement containing detailed terms and conditions of use. Patient-level datasets can be extracted for researchers against specific study specifications, following protocol approval from the Independent Scientific Advisory Committee (ISAC).” Thus, restrictions apply to the availability of these data, which were used under license for the current study and are not publicly available.
