# Supplementary material for: Stratification of diabetes in the context of comorbidities, using representation learning and topological data analysis
Source: Sci Rep. 2023 Jul 16;13:11478. doi: 10.1038/s41598-023-38251-1 (PMC10350454; doi:10.1038/s41598-023-38251-1)
Supplement: Supplementary file 1 — Supplementary Information 1. [file 41598_2023_38251_MOESM1_ESM.pdf]

Supplementary material

1. Data and study population

This study has been performed on a CPRD Gold cut containing 4 million patients registered between 1985 and 2015 with linkage to HES (Hospital Episode Statistics) for secondary care records and ONS for death register records.

To fully cover patients' health journeys, only patients with hospital and ONS data linkage were included in the study. Each practice in CPRD has a date after which data was up-to-standard; only events recorded after this date were included in the study.

In this study, we are interested in patients with new onsets of diabetes mellitus (DM), where DM is defined as per its CALIBER code. To have broad coverage of patients' medical history and similarly to BEHRT (4), only subjects with at least 5 visits in their records are considered in this study. Moreover, to provide our models with enough past events to learn from, we included only those subjects whose diagnosis of DM was registered at least as nth comorbidity (i.e. a DM occurrence is only considered if at least n-1 other diseases were reported before it). In this paper, we used n=7, which is large enough to enable the model to learn from enough past events and still produce a significant number of patients. This results in 9,967 patients with diabetes mellitus (DM).

In traditional research using EHR data (including the ones using ML), experts define several features/markers at a given baseline to represent the patients. Recent developments in DL, however, provided us with models that can learn optimal representations (e.g., of individuals or diseases) from raw or minimally processed data, with minimal need for expert guidance (and hence the name representation learning). The complexities of EHR data, which can result in a vast universe of possible representations, led to the growing popularity and superior performance of representation learning in this field.

2. BEHRT

To extract contextualised representations from EHR, we used the BEHRT architecture, which has been shown to achieve state-of-the-art performance in predicting the onset of new diseases (4). In addition to diagnoses, medications, and age, BEHRT uses encodings for positions (counter of the visit) and segments (to distinguish between consecutive visits). At any holiday, the sum of all these embeddings will result in an embedding (shown at the bottom of Figure 1S (I)), which will be the learned representation of one's EHR at that point.

Figure 1S shows BEHRT's Transformer-based architecture. It is first pre-trained by the masked language model (MLM) task (similar to the original BERT architecture (5)), to learn the network parameters (including the disease and drug embeddings) that can predict the masked disease tokens. When used for downstream tasks such as disease predictions, the model finetunes the weights pre-trained in the MLM task and learns the weights for the classification layer.

In this study, we used the embeddings obtained using the MLM task (shown across layers from $\{E_i\}$ to $\{T_i\}$ vectors, in Figure 1S (II)) to have a contextualised representation of one's EHR at a given point in time which is not biased towards a particular clinical outcome. Note that, the closer we get to the T_i layer (i.e., further away from the E_i layer), the more contextualised the embeddings are likely to be (6). Therefore, we expect (i.e., our hypothesis) to observe different embeddings for the same token (i.e. a given disease or medication) due to differences in their surrounding sequence (i.e., context).

Note that to avoid any potential leakage, we define for each patient, the baseline t as the date of occurrence of DM (as n^{th} comorbidity) and will only feed to BEHRT the encounters which occurred on or before t. To feed every included patients' EHR to BEHRT – given the inconsistencies in their length – we will pad the records that are shorter than the required length (fixed at 256 tokens in this study).

This architecture has been shown to capture a context-aware representation of words/tokens in the Natural language processing field (6). The aim of this study was to show that these embeddings provide an accurate

representation of the current state of every patient' health condition and that they can be used to mine different patterns of a given disease.

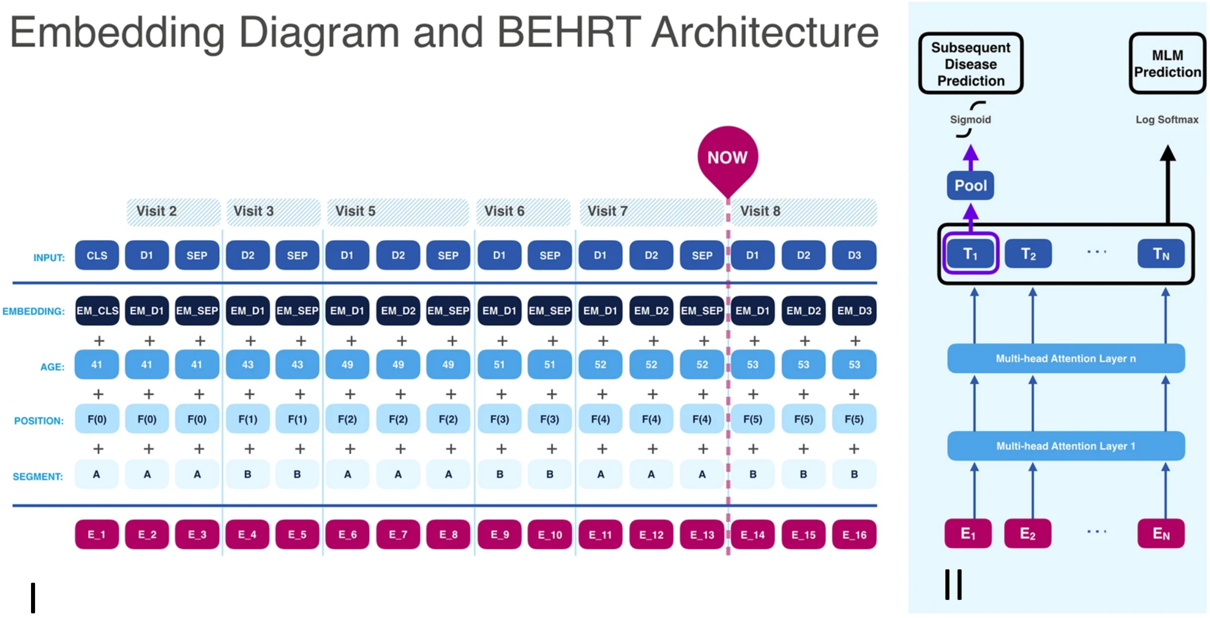

Figure 1S: Encoding EHR data using BEHRT (credits to (4)).

3. Topological data analysis

In this study, we used a TDA technique called Mapper (7), which operates as follows: a) it projects the data from the embedding space to a lower dimensional space, b) it covers the data in the projection space using overlapping hypercubes, c) it performs clustering of the projected data within each hypercube. Each obtained cluster will correspond to a node in the graph, if a data point belongs to more than one cluster, these clusters will be linked using an edge. Figure 2S illustrates this process. We used a Python implementation of Mapper called Kepler Mapper (8,9).

In this study we used t-SNE as our mapping space as it has been shown to perverse many existing relationships in a lower dimensional space (10). This has been done using the following parameters set empirically: we used K-Means as a clustering algorithm with 3 clusters and 20 hypercubes with a 50% overlap proportion. These parameters have been set to obtain a fully connected graph network structure; changing these parameters will generally not substantially alter the structure of the graph; however significantly reducing the overlap proportion will results in disjoint systems. Similarly, significantly reducing the number of hypercubes will result in a very small chart which may not be useful for untangling complex relationships. It is, therefore generally necessary to fine-tune the parameters of the TDA algorithm until the obtention of a detailed and succinct description of the data (11).

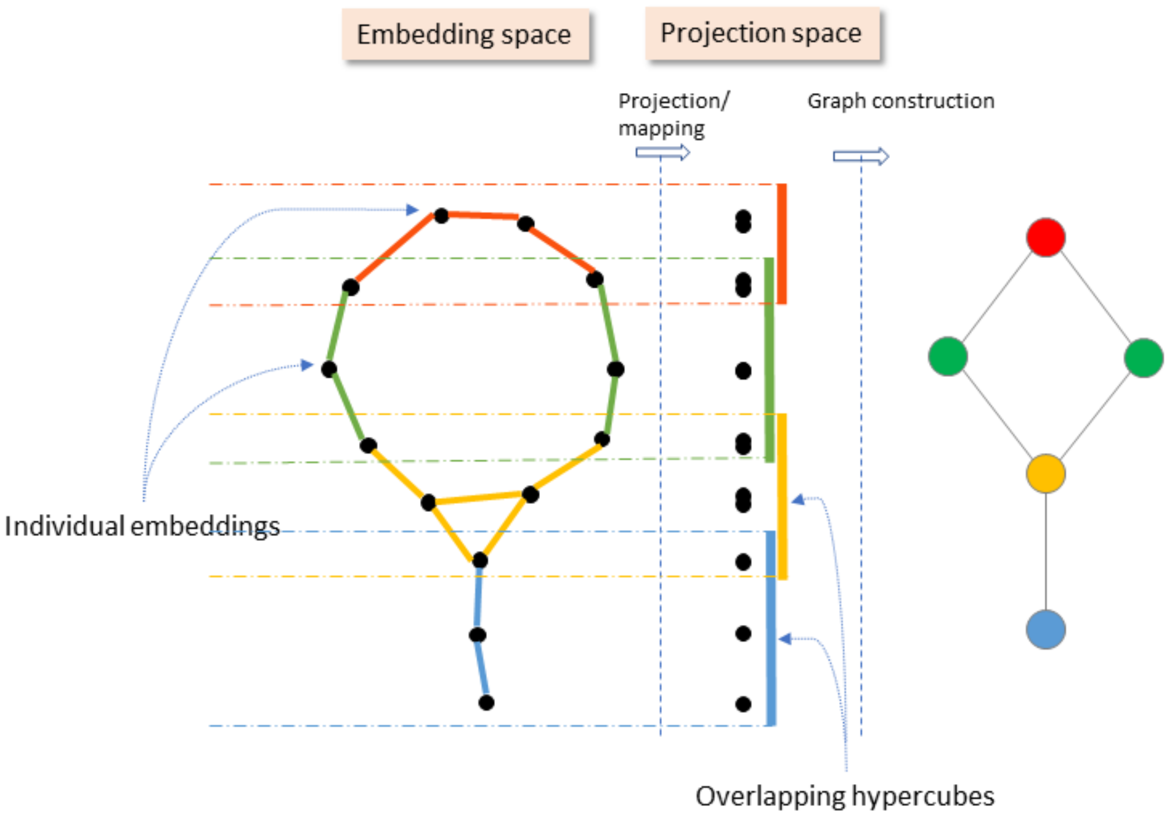

Figure 2S: Illustration of TDA Mapper algorithm

4. Graph partitioning method

The obtained TDA graph is partitioned using the multilevel k-way partitioning algorithm, which cuts the graph size by collapsing its nodes and edges (coarsening step), finds a k-way partition of the condensed graph. Then it constructs a k-way partition for the original graph by projecting and refining the partition to successively finer graphs (uncoarsening step) (12,13). In this study, we used the implementation provided by the METIS graph partitioning software (14).

This algorithm segments the graph into *n* contiguous regions. To determine the optimal value of *n*, we increment it until we reach the value for which the difference between the top diseases between each pair of obtained clusters is not significant anymore (P-values of the corresponding Chi-squared test greater than 0.05).

5. Handling missing data

Since the data is not necessarily missing at random and may be related to the unobserved values themselves, imputing missing data may not be the optimal practice, as it may introduce bias and affect the validity of the summary statistics. For this reason, only patients with complete data are considered when producing counts for variables with missing data (such as BMI). It is worth noting that transformers, like sequence-to-sequence models, do not have fixed input or output format or length when developing the model architecture. They take a sequence of varying lengths and output a sequence of varying lengths, making missing data irrelevant. All information becomes a vector in a sequence of vectors. Thus, the method of including only cases without missing data does not challenge the model architecture.

References

1. Read Codes - NHS Digital [Internet]. [cited 2021 Jan 17]. Available from: https://digital.nhs.uk/services/terminology-and-classifications/read-codes

2. Booth N. What are the Read Codes? Health Libr Rev [Internet]. 1994 [cited 2021 Jan 17];11(3):177–82. Available from: https://pubmed.ncbi.nlm.nih.gov/10139676/

3. Kuan V, Denaxas S, Gonzalez-Izquierdo A, Direk K, Bhatti O, Husain S, et al. A chronological map of 308 physical and mental health conditions from 4 million individuals in the English National Health Service. Lancet Digit Heal [Internet]. 2019 Jun 1 [cited 2021 Jan 17];1(2):e63–77. Available from: www.thelancet.com/

4. Li Y, Rao S, Solares JRAJRA, Hassaine A, Canoy D, Zhu Y, et al. BEHRT: Transformer for Electronic Health Records. Sci Rep [Internet]. 2020 [cited 2020 Feb 26];10(1):1–12. Available from: https://www.nature.com/articles/s41598-020-62922-y

5. Devlin J, Chang M-W, Lee K, Toutanova K. BERT: Pre-training of Deep Bidirectional Transformers for Language Understanding. 2018 Oct 10 [cited 2020 Feb 12]; Available from: http://arxiv.org/abs/1810.04805

6. Akbik A, Bergmann T, Vollgraf R. Pooled contextualized embeddings for named entity recognition. In: Proceedings of the 2019 Conference of the North American Chapter of the Association for Computational Linguistics: Human Language Technologies, Volume 1 (Long and Short Papers). 2019. p. 724–8.

7. Singh G, Mémoli F, Carlsson G. Topological Methods for the Analysis of High Dimensional Data Sets and 3D Object Recognition. Eurographics Symp Point-Based Graph. 2007;

8. van Veen HJ, Saul N, Eargle D, Mangham SW. Kepler Mapper: A flexible Python implementation of the Mapper algorithm [Internet]. Zenodo; 2020. Available from: https://doi.org/10.5281/zenodo.4077395

9. van Veen HJ, Saul N, Eargle D, Mangham SW. Kepler Mapper: A flexible Python implementation of the Mapper algorithm. J Open Source Softw [Internet]. 2019;4(42):1315. Available from: https://doi.org/10.21105/joss.01315

10. Maaten L van der, Hinton G. Visualizing data using t-SNE. J Mach Learn Res. 2008;9(Nov):2579–605.

11. Dagliati A, Geifman N, Peek N, Holmes JH, Sacchi L, Bellazzi R, et al. Using topological data analysis and pseudo time series to infer temporal phenotypes from electronic health records. Artif Intell Med. 2020 Aug 1;108:101930.

12. Karypis G, Kumar V. Multilevel k-way partitioning scheme for irregular graphs. J Parallel Distrib Comput. 1998;48(1):96–129.

13. Karypis G, Kumar V. Parallel multilevel k-way partitioning scheme for irregular graphs. 1996 [cited 2022 May 5];35-es. Available from: http://www.cs.umn.edu/˜karypis

14. Karypis G, Kumar V. METIS: A Software Package for Partitioning Unstructured Graphs, Partitioning Meshes, and Computing Fill-Reducing Orderings of Sparse Matrices. 1997 [cited 2022 Jan 20]; Available from: http://conservancy.umn.edu/handle/11299/215346
